# An autoregulatory loop reverts the mechanosensitive *Sirt1* induction by EGR1 in skeletal muscle cells

**DOI:** 10.18632/aging.100470

**Published:** 2012-07-18

**Authors:** Patricia S. Pardo, Aladin M. Boriek

**Affiliations:** Department of Medicine, Division of Pulmonary, Critical Care and Sleep, Baylor College of Medicine; Department of Cell and Molecular Biology, Baylor College of Medicine, Houston, TX 77030

**Keywords:** Mechanotransduction, oxidative stress, skeletal muscles, Sirt1

## Abstract

Muscle contraction is associated with the production of reactive oxygen species (ROS). Mechanisms of ROS scavenging are fundamental to avoid muscle damage. We had previously discovered a stretch-induced genetic program in myotubes that triggers an antioxidant defense. At the core of this mechanism, transcriptional activation of SIRT1 by the early growth response protein EGR1 results in increased MnSOD activity through the activation of *Sod2* by SIRT1/FOXO pathway. In this report, we show experimental evidence that; a) EGR1 and SIRT1 proteins physically interact at the time of maximal *Sirt1* induction, b) SIRT1 has a negative effect on the activation of the *Sirt1* promoter by EGR1. Thus, the interaction between EGR1 and SIRT1 describes an autoregulatory loop that shuts down the stretch-induced *Sirt1* expression.

## INTRODUCTION

SIRT1 (sirtuin 1) is a protein deacetylases that uses NAD+ as a cofactor in such a way that their activity is modulated by redox status. In skeletal muscle cells, SIRT1 activity plays important roles in differentiation and adaptations to nutrient availability and exercise [reviewed in 1]. Several lines of evidence have shown that SIRT1 plays a major role in mechanisms of antioxidant defense in several tissues [2-5]. Contraction associated ROS production results in cumulative oxidative stress in skeletal muscles [6]. Recent findings from our group implicated EGR1 and SIRT1 in a mechanical stretch-induced mechanism that contributes to ROS detoxification. In particular, a fast induction of the *Sirt1* gene occurs in myotubes in response to mechanical stretch [7]. The up-regulation of SIRT1 expression by stretch occurs at the transcriptional level and requires the binding of the early growth response protein, EGR1 to the *Sirt1* promoter. The stretch-dependent induction of *Sirt1*, leads to SIRT1-dependent FOXO4 deacetylation and increased expression of its target, the Mn-dependent superoxidase dismutase gene, *Sod2* [7, 8]. This stretch-induced gene activation program contributes in the removal of the excess of reactive oxygen species, ROS, generated by the mechanical stimulus and serves to prevent long exposure to ROS levels that might result in cell damage.

The transcriptional activation of *Sirt1* by stretching skeletal myoctes is a transient mechanism, changes in SIRT1 RNA and protein expression and their downstream effects occur in a short lapse, between 2 and 8 hours after stretch. To unravel the mechanism responsible for shutting down the stretch-induced *Sirt1* gene activation, we explored the possibility of the existence of an autoregulatory loop involving physical and/or functional interactions between SIRT1 and EGR1. Negative feedback loops between SIRT1 and its transcriptional regulators have been previously described for c-Myc [9] and E2F1 [10].

## RESULTS

### Stretched-induced SIRT1 interacts with EGR1 in C2C12 myotubes

**Figure 1:**
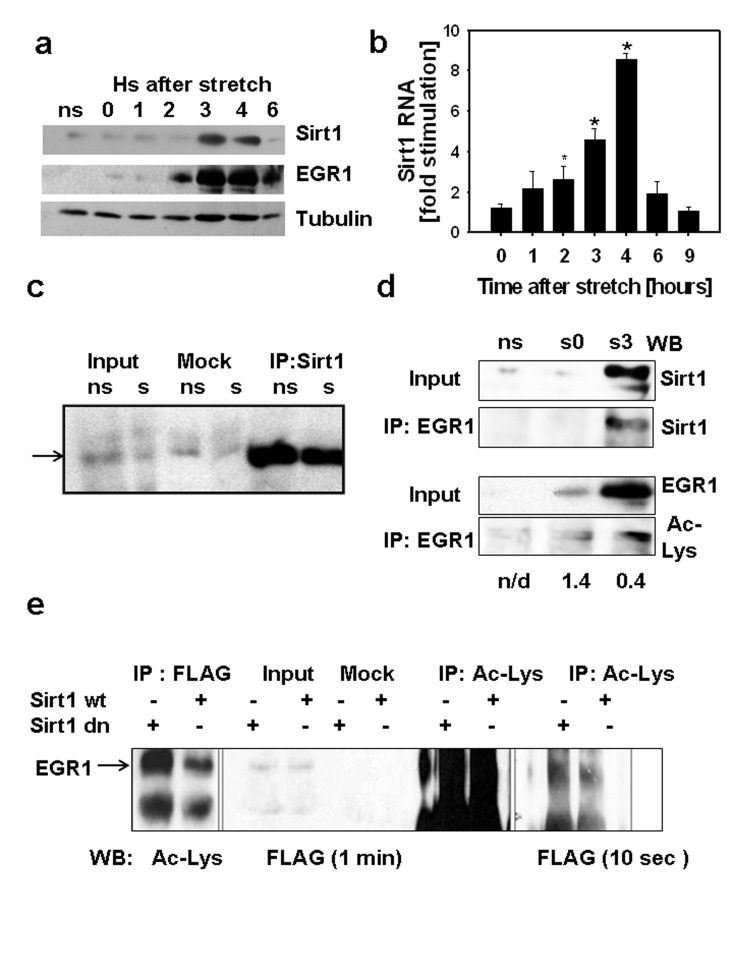
(**a**) C2C12 myotubes were cultured on 7 FlexCell plates, 6 plates were subjected to 30 min stretch while a set of myotubes was kept without stretch (ns), stretched myotubes were harvested at different times after stretch from 0 to 6 hours. 80 μg of total protein from each sample and molecular weight markers (MW) were subjected to SDS-PAGE and Western blot with antibodies against SIRT1, EGR1 and tubulin. Molecular weight markers positions are displayed on the left of the western blot scan images. (**b**) SIRT1 RNA content was determined by RT-real time PCR in stretched and non-stretched myotubes by RT-real time PCR and fold stimulation represents the stretched/non-stretched SIRT1 RNA content ratios (*indicates statistical significant difference from non-stretched) (**c**) Stably expressing FLAG-EGR1C2C12 cells (C2C12 12.4 clone) were plated in flexible bottomed plates and a set of cells were stretched (s) whereas another set was used as non-stretched controls (ns). Total proteins were obtained 3 hours after stretch; 0.5 mg of protein were incubated with protein A/G agarose beads loaded with a rabbit SIRT1 antibody or without antibody (mock); 50μg of total protein (input) and mock and SIRT1 immunoprecipitates were subjected to SDS-PAGE and Western blot with a monoclonal anti-FLAG M2 peroxidase conjugated antibody (Sigma-Aldrich). (**d**) Total proteins (0.4 mg) from non-stretched myotubes (ns), myotubes subjected to 30 min stretch and harvested immediately (s0) or 3 hours after (s3) were immunoprecipitated with anti-EGR1, 75 μg of total protein (input) and immunoprecipitates from each sample were analyzed by Western blot with a mouse monoclonal anti-SIRT1 (Sigma-Aldrich) or a mouse monoclonal anti-acetyl lysine (Upstate). The numbers below each lane represent the estimate ratio of acetylated EGR1.total EGR1 obtained by densitometric analysis of the films. (**e**) 0.75 mg of total protein from C2C12 12.4 cells transfected with pYE-Sir2 or pYE-Sir2 (H/Y) were incubated overnight without (mock) or with a rabbit polyclonal anti-acetyl lysine antibody and immunoprecipitated with A/G agarose beads; 80ug of total protein and total immunoprecipitates were subjected to Western blot with anti-FLAG M2 peroxidase, images of the film exposed for 1 min (right) or 10 sec(left) are shown.

In C2C12 myotubes subjected to a 30 min lapse of mechanical stretch, EGR1 protein content increased immediately after the application of stretch, peaked between 3 and 4 hours after stretch and diminished rapidly in the next 2 hours (Fig1a and Fig 1S). The induction of SIRT1 protein by stretch requires transcriptional activation of the *Sirt1* promoter by EGR1 [7]. SIRT1 RNA and EGR1 protein induction peaked between 3 and 4 hours (Fig. 1a and 1b). This suggests the existence of transcriptional and post-transcriptional mechanisms which actively down-regulate SIRT1 expression. To explain the downregulation of *Sirt1* transcription, we considered the possibility that at the time of maximal SIRT1 protein expression, physical and/or functional interactions between SIRT1 and EGR1 trigger mechanism/s which downregulate SIRT1 transcription after stretch. For this purpose, we generated a C2C12 cell lineage, C2C12 12.4, stably expressing an EGR1-FLAG fusion protein.

Physical association between EGR1 and SIRT1 was explored in myotubes obtained from C2C12 12.4 myoblasts. In co-immunoprecipitation experiments, SIRT1 was immunoprecipitated and EGR1 was detected with an anti-FLAG antibody in stretched and non-stretched myotubes (Fig.1c). The association between endogenous EGR1 and SIRT1 proteins was evaluated by co-immunoprecipitation in non-stretched and stretched myotubes, immediately after or three hours after stretch. SIRT1 was detected on EGR1 immuno-precipitates three hours after stretch (Fig. 1d). These results indicate that a SIRT1- EGR1 physical association is possible and effectively occurs when EGR1 and SIRT1 proteins reach maximal expression after stretch. The observation that in C2C12 12.4 myotubes the association between EGR1 and SIRT1 was not affected by stretch suggests that interaction between those two proteins depends on their level of expression rather than other possible stretch-dependent signal.

Considering that EGR1 can be modulated by acetylation [11], we analyzed the state of acetylation of EGR1 by western blot with acetyl lysine antibodies on EGR1 immunoprecipitates before and after stretch (Fig. 1d).These data suggest that the change in the expression of EGR1 during the 3 hours after stretch does not correlate with a similar change in the state of acetylation. For a semi-quantitative estimation, the relative content of acetylated EGR1 to total EGR1 was estimated by densitometric analysis of the western blots. The acetylated EGR1/total EGR1 ratios calculated (shown in Fig.1 d) indicates that at least, a 66% reduction in the proportion of acetylated EGR1 occurs after stretch.Then we evaluated the ability of SIRT1 to deacetylate EGR1 by assessing the state of acetylation of EGR1 in C2C12 12.4 cells overexpressing SIRT1 or a dominant negative SIRT1 mutant. Immuno-precipitation of EGR1 or acetylated proteins showed an increase in acetylated EGR1 when the dominant negative SIRT1 was expressed (Fig. 1e) suggesting that SIRT participates in EGR1 deacetylation.

### SIRT1 supresses the EGR1-dependent activation of the Sirt1 promoter

**Figure 2:**
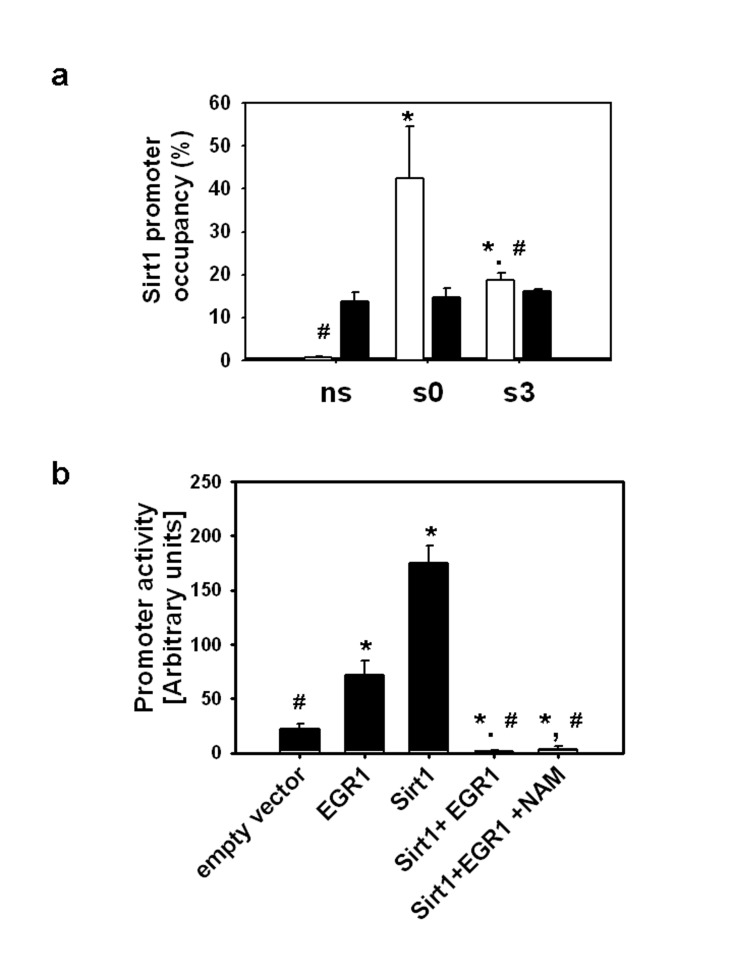
(**a**) ChIP assays were performed with EGR1(▭) and SIRT1 (▬) antibodies on non-stretched myotubes (ns), myotubes subjected to 30 min stretch and harvested immediately (s0) or 3 hours after (s3), immunoprecipitated DNA was analyzed by real time PCR with primers for the EGR1 binding sites and promoter occupancy was estimated as described in Methods. * and # indicates statistical significant difference from ns or s0, respectively. (**b**) C2C12 myoblasts grown on 24 well plates were transfected with the Sirt1 promoter reporter (0.1 μg/well) in the absence or presence of pcDNA-EGR1 and/or pcDNA-SIRT1 (0.2 μg/well); pcDNA was incorporated in the DNA mixtures to complete 0.5 μg/well and nicotinamide (NAM) to a 10 mM final concentration was added to the media when indicated. Luciferase activity was determined 24 hours after transfection with Dual Glo luciferase (Promega), * and # means statistical significant difference from empty vector or EGR1, respectively.

To evaluate if SIRT1 interacts with EGR1 on the *Sirt1* promoter, ChIP assays were performed with a SIRT1 antibody followed by amplification of the region encompassing the EGR1 binding sites. As seen in Fig. 2a, EGR1 binding to the *Sirt1* promoter is higher immediately after stretch and decays at the time EGR1 reaches maximal induction, whereas SIRT1 binding to the same region remains unaffected. These data indicate that 1) the SIRT1 protein does not interact with EGR1 on its consensus binding sites on the Sirt1 promoter, 2) EGR1 binding to the *SIRT1* promoter does not passively respond to changes in EGR1 levels.

In order to establish the effect of SIRT1 protein overexpression on EGR1-driven *Sirt1* transcription, C2C12 cells were transiently co-transfected with the Sirt1 promoter luciferase reporter and pcDNA-EGR1-FLAG, pcDNA-SIRT1-FLAG or both. The *Sirt1* promoter was activated by EGR1 or SIRT1 independently, whereas overexpression of both proteins completely suppressed the activity of the promoter. Addition of the SIRT1 inhibitor, nicotinamide, to the media did not alter the inhibitory effect of SIRT1 on EGR1-dependent activation of the *Sirt1* promoter (Fig. 2b). These combined data suggest that increased expressions of EGR1 and SIRT1 are necessary and sufficient for the suppression of the EGR1-dependent activation of the *Sirt1* promoter whereas the SIRT1 activity and consequent EGR1 deacetylation are not necessary.

Taken together, the results presented in this report suggest that an increase in SIRT1 expression favors the physical interaction between EGR1 and SIRT1 and results in the suppression of the EGR1-dependent activation of the *Sirt1* gene possibly by preventing EGR1 binding to the Sirt1 promoter.

## DISCUSSION

Muscle contraction increases generation of ROS under physiological conditions and has been associated with diminished contractile function and fatigue during exercise [12]. In the long term, ROS production leads to cell damage: increased oxidative stress due to altered redox status is a hallmark of skeletal muscle aging [1, 5, 13]. Thus, a better understanding of the mechanisms controlling anti-oxidative defense in skeletal muscle cells in response to mechanical stimuli may help to define proper interventions to prevent the deleterious effects of oxidative stress on muscle function at old ages.

We have previously reported that stretch induced transcriptional activation of *Sirt1* through EGR1 leads to the FOXO-dependent activation of the *Sod2* gene and the consequent increased MnSod activity promotes ROS scavenging [7].

Interestingly, our published data showed that the stretch-induced activation of *Sirt1* is rapidly turned off. SIRT1 expression is regulated at multiple levels [14]. Posttranscriptional mechanisms control SIRT1 transcript stability through RNA binding proteins and microRNAs which are responsible for maintaining basal SIRT1 levels in embryonic cells and adult tissue [15-17]. At the transcriptional level, the involvement of SIRT1 in the control of its own expression by controlling the activity of its transcription regulators appears to be a common mechanism in situations of acute stress conditions as genotoxic stress, hypoxia or nutrient deprivation [9, 18].

**Figure 3:**
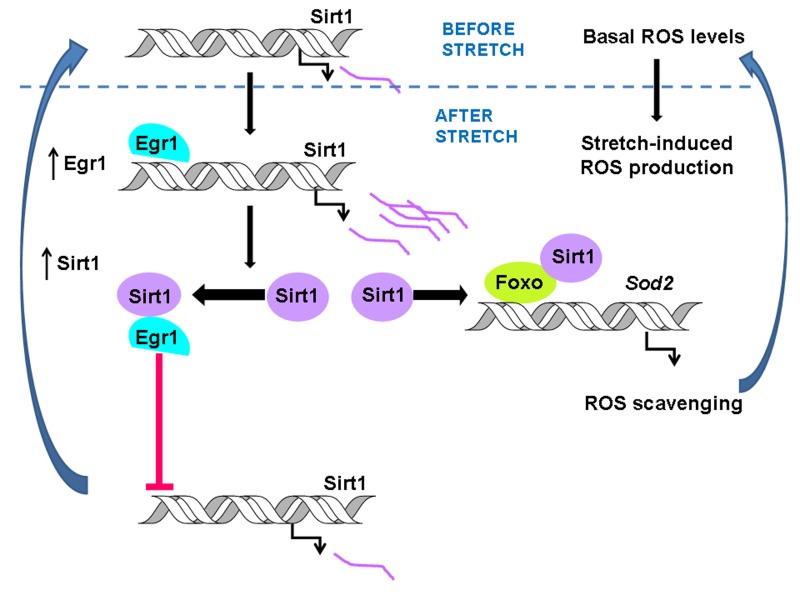
The scheme summarizes the stretch-induced pathway that allows ROS scavenging in response to mechanical stimuli. As previously described, stretch–dependent transcriptional activation of Sirt1 by EGR1 activates the Sod2 gene by stimulating FOXO binding to the Sod2 promoter leading to lowering ROS content to its basal levels [6]. The interaction between EGR1 and SIRT1 prevents EGR1 binding to the Sirt1 promoter triggering an autoregularoty loop that turns down SIRT1 expression from the stretch-induced to basal levels.

In this report, we evaluated whether a physical or functional interaction between EGR1 and SIRT1 protein was responsible for shutting down the induction of *Sirt1* by EGR1 in stretched skeletal muscle cells. Our results point out that EGR1 and SIRT1 have the ability to interact when artificially overexpressed, and that this physical interaction occurs when both proteins nearly reach maximal expression after stretch in myotubes. The analysis of the *Sirt1* promoter activity showed that SIRT1 protein overexpression prevents the positive effect of EGR1 on the *Sirt1* promoter. Unexpectedly, SIRT1 had a positive effect on its own promoter activity in the absence of EGR1, which discards the possibility that repression occurs by SIRT1 interaction with an alternative transcriptional modulator. Though, our *in vitro* results suggest that SIRT1 has the ability to deacetylate EGR1, SIRT1 activity was dispensable for the inhibition of EGR1-driven transcription of the *Sirt1* promoter. The time course analysis of the binding of EGR1 to the *Sirt1* promoter after stretch showed that EGR1 binds to the Sirt1 promoter early after stretch but not at the time when both, EGR1 and SIRT1 reach maximal levels of expression. These data provide experimental evidence of a negative loop mechanism by which, at the time maximal induction of EGR1 and SIRT1 proteins occurs, their physical interaction is responsible for switching off the stretch-induced *Sirt1* expression by precluding EGR1 from binding to the *Sirt1* promoter as summarized in the scheme on Figure 3. Through the negative feedback mechanism described here, at the time ROS content recovered their basal levels, EGR1 and SIRT1 protein contents also return to their initial state. Thus, this mechanism allows cells to respond to further stretch signals and to maintain ROS homeostasis.

## MATERIALS AND METHODS

### Materials

pcDNA3-EGR1 –FLAG [19], pTA-SIRT1 promoter (-202) [20], pYE-Sir2 and pYE-Sir2(H/Y) were obtained from Addgene. pTA-Luc was obtained from CLontech. pcDNA-SIRT1 –FLAG [21] was a kind gift from Dr. Gu. Antibodies were from Millipore except M2 from Sigma Aldrich and EGR1 (C-19) from Santa Cruz Biotechnology Inc.

### Cell lines and culture

C_2_C_12_ cells were obtained from ATCC. Myoblasts were grown in high glucose DMEM media (Invitrogen) supplemented with 10% FBS and 0.5 mg/ml of minimal essential aminoacids (GM) in a CO_2_ incubator. Myotubes were obtained from myoblasts grown at subconfluency by replacing the media by high glucose DMEM supplemented with 2% horse serum (DM) after a wash with phosphate base saline (PBS). 70-80% of myotubes are obtained at 2nd day on regular collagen coated plates (Biocoat) or at 3rd day on type I collagen coated flexible bottomed plates (Flex I type Flexcell) Stable clones expressing EGR1-FLAG were obtained by transfection of pcDNA3-EGR1 with Fugene (Roche) on C2C12 myoblasts; after 48 hrs of transfection, cells were split 1:10 and allowed to grow in GM in the presence of 500 μg/ml of gentamycin for 10 days. Isolated colonies were identified by microscopy observation and the cells from each clone were recovered by treatment with 0.1 mg/ml of trypsin under the pipette tip. Trysin treated colonies were plated in 60 mm plates in GM with 100 μg/ml of gentamycin. Expression of EGR1-FLAG was evaluated by Western blot with an M2 antibody tagged to peroxidase. A highly expressing clone C_2_C_12_ 12.4 was expanded and aliquots were kept frozen.

### Myotubes stretch protocol

Myoblasts cultured onto type I collagen-coated flexible-bottom wells (Flex I plates, FlexCell International) were subjected to differentiation. At third day in DM, myotubes were subjected to a 15% stretch protocol at 1 Hz (0.5 s of deformation alternating with 0.5 s of relaxation) using a Flexcell system. Myotubes were stretched for 30 min and harvested at different intervals after stretch.

### Protein extraction, immunoprecipitation and Western blot

Cultured myotubes were washed in cold PBS and scrapped. Cell pellets were resuspended in a cell lysis buffer (Tris HCl pH 7.5 25 mM, NaCl 250 mM, EDTA 2 mM, Nonidet P40 0.3%) containing PMSF 0.5 mM, DTT 1mM and protease inhibitors. After 2 cycles of freeze and thaw, extracts were microcentrifuged for 10 min at 10000 rpm and pellets were discarded. Protein concentration was determined with Quick Start Protein from BioRad. Indicated amounts of total protein were denatured in SDS-PAGE sample buffer, analyzed in 10% SDS-PAGE gels and transfer to nitrocellulose membranes. For immunoprecipitation, 400-750 μg of whole protein extract was diluted with an equal volume of Tris HCl 7.5 25 mM containing protease inhibitors and were pre-cleared by incubation for 1 h with 50 μl of A/G agarose beads in a rotor. Pre-cleared extracts were incubated overnight with 1 μg of the indicated antibodies by each 100 μg of protein. Mock samples were incubated without antibody. A/G agarose beads (5 μl/μg of antibody) were added by 3 hours, washed 3 times with phosphate buffer saline (PBS) for 10 min and analyzed by Western blot.

### RNA extraction and real time PCR

Total RNA from cells was prepared with the RNAeasy mini kit from Qiagen. Total RNA from diaphragm muscles was prepared with Trizol (Invitrogen). Reverse transcription was performed on 1 μg of total RNA. cDNA aliquots were amplified with the Brilliant Sybr Green Master Mix (Stratagene) in a MX3005P cycler (Stratagene). RNA contents were standardize for GAPDH with the formula 2^−Ct × −Ct GAPDH^. Primers sequence will be provided upon request.

### Luciferase assays

C2C12 myoblasts were co- transfected with either pTA-Luciferase or pTA-Sirt1 promoter and a hRLuc-TK vector (pGL4. 74 from Promega) keeping a 10:1 ratio. When indicated pcDNA-EGR1 and pcDNA-SIRT1 were co-transfected. Transfections were performed with Lipofectamine 2000 (Invitrogen). Assays were performed with the Dual Glo Luciferase kit (Promega).

### ChIP assays

C2C12 myotubes were subjected to the indicated treatments and then fixed in DM containing 1% formaldehyde for 10 min. Crosslinking was stopped by adding glycine to a final concentration of 0.125 M followed by 5 min incubation at room temperature. The cells were washed with PMSF 0.05 mM in PBS twice and scrapped. EZ-CHIP kit was used following manufacturer instructions. Cell pellets were suspended in SDS Lysis Buffer containing 0.5 mM PMSF and 2x protease inhibitor cocktail and sonicated (8 strokes at 30% for 15 sec followed by 15 sec rest) while kept in a ice-water bath. After 5 times dilution in CHIP dilution buffer, 10 μl were separated (input) and the remaining was incubated with the indicated specific antibody or rabbit IgG(negative control). Immunoprecipitated DNA was analyzed by real time PCR with the following primers Fw: GCGTGGGTGGCGG GAGAGG and Rv: CATCTTCCAACTGCCT CTCTGGCC. Promoter occupancy was calculated using the formula: 2 ^−Ct IP-Ct input −2−Ct negative control −Ct input^.

### Statistics

Data from RNA and ChIP assays are expressed as means ± SD obtained from three independent experiments. Luciferase assays were performed by quadruplicate and plotted data are means ± SD from two independent experiments. Statistical significance was analyzed by one-way ANOVA with a pairwise comparison by Holman-Sidak with P < 0.05.

## Abbreviations

ROS: reactive oxygen species
SIRT1: sirtuin 1
EGR1: early growth response protein 1
FOXO: Forkhead box protein O
SOD2: Superoxide dismutase 2
MnSOD: manganese-dependent superoxide dismutase
